# Comparative Analysis of Phosphoproteome Remodeling After Short Term Water Stress and ABA Treatments versus Longer Term Water Stress Acclimation

**DOI:** 10.3389/fpls.2017.00523

**Published:** 2017-04-11

**Authors:** Govinal B. Bhaskara, Thao T. Nguyen, Tsu-Hao Yang, Paul E. Verslues

**Affiliations:** Institute of Plant and Microbial Biology, Academia SinicaTaipei, Taiwan

**Keywords:** phosphoproteomics, drought stress, abscisic acid (ABA), abiotic stress, Arabidopsis proteins, phosphorylation, phos-tag electrophoresis, osmotic stress

## Abstract

Several studies have used short term dehydration, osmotic stress or Abscisic Acid (ABA) treatments to identify the initial protein phosphorylation-dephosphorylation responses to drought and low water potential or ABA treatments. However, longer term drought acclimation leads to altered expression of many kinases and phosphatases suggesting that it may also produce unique changes in phosphoproteome composition. To get a better overview of the state of drought-related phosphoproteomics and investigate this question of short versus longer term phosphoproteome regulation, we compared three *Arabidopsis thaliana* studies analyzing short term phosphoproteome changes to recent data from our laboratory analyzing phosphoproteome changes after a longer drought acclimation treatment. There was very little overlap of phosphoproteins with putative stress-induced phosphorylation or dephosphorylation among these studies. While some of this is due to technical limitations and limited coverage of the phosphoproteome achieved by each study, biological differences and the type of stress treatment used also play a role. This comparative analysis emphasized how both short and long term analysis of physiologically relevant stress treatments, as well as validation of phosphoproteomic data, will be needed to move past just scratching the surface of the stress phosphoproteome. In drought acclimation experiments, distinguishing between changes in protein abundance versus phosphorylation stoichiometry is a key challenge. We discuss initial work in using Arabidopsis seedling transient expression combined with Phos-tag gel analysis as a way to validate drought-induced phosphorylation-dephosphorylation of candidate proteins.

## Introduction

Post-translational modification of proteins by phosphorylation/dephosphorylation is a key element of low water potential and drought response as well as hormone signaling. Plants have greatly expanded numbers of protein kinases compared to vertebrates. The Arabidopsis genome contains more than 1,000 kinases (Wang et al., 2007; Lehti-Shiu and Shiu, 2012). Most of these kinases are of unknown function (Schweighofer et al., 2004; Ding et al., 2009). Phosphatases are nearly 10-fold less in number (112–164 in different analyses of the Arabidopsis genome, Wang et al., 2007; Yang et al., 2010), but similar to kinases, most are still of unclear function (Fuchs et al., 2013). Perhaps the most prominent example of the key role of kinase-phosphatase signaling in abiotic stress is the core abscisic acid (ABA) signaling pathway formed by Clade A Protein Phosphatase 2Cs (PP2Cs), the PYL/RCAR family of ABA receptors/phosphatase regulators and Sucrose non-fermenting Related Kinase2 (SnRK2) kinases (Cutler et al., 2010; Raghavendra et al., 2010). However, even for the relatively well characterized SnRK2 kinases, phosphoproteomics analysis has demonstrated that they have many yet uncharacterized phosphorylation targets (Umezawa et al., 2013; Wang et al., 2013). These data, together with the large number of uncharacterized kinases in Arabidopsis and many potential protein phosphorylation sites in data bases such as PhosPhAt (Durek et al., 2010)^1^, indicate that a great number of abiotic stress- and ABA-regulated phosphorylation-dephosphorylation sites remain to be experimentally identified.

**FIGURE 1:**
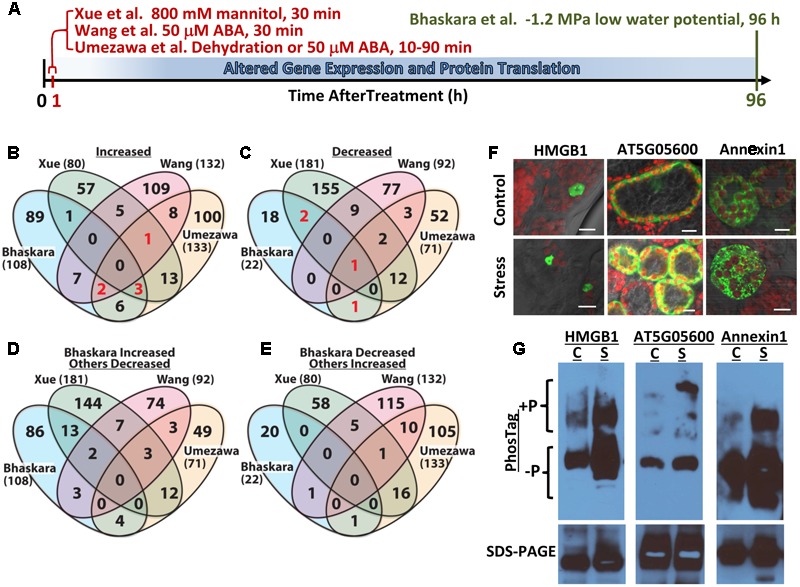
**Comparative analysis of four abiotic stress and ABA-related phosphoproteomic datasets and use of seedling transient expression and Phos-tag protein separation for exploratory analysis of phosphoproteins. (A)** Diagram showing the different treatments and times of sample collection in the four phosphoproteomic studies compared here. Umezawa et al. (2013) analyzed 10–90 min dehydration or 50 μM ABA treatments and used a 3-fold cutoff to define phosphopeptides of altered abundance. Wang et al. (2013) used 50 μM ABA treatment for 30 min and used a 2-fold cutoff to define differentially regulated phosphopeptides. Xue et al. (2013) used 30 min treatment with 800 mM mannitol and a standard curve based approach combined with a 2-fold change cutoff to identify phosphopeptides of significantly altered abundance. Bhaskara et al. (2017) used 96 h low water potential treatment and *P* ≥ 0.05 combined with a 1.5-fold change cutoff. **(B)** Venn diagram comparing proteins with one or more phosphopeptide of increased abundance in stress or ABA treatment for all four datasets. Each data set is identified by the name of the first author and numbers in parentheses are the total number of proteins with increased phosphopeptide abundance in that dataset. Red numbers indicate phosphoproteins included in **Table 1**. **(C)** Comparison of proteins with one or more phosphopeptide of decreased abundance in stress or ABA treatment for all four datasets. Red numbers indicate phosphoproteins included in **Table 1**. **(D)** Converse comparison of proteins with increased phosphopeptide abundance in Bhaskara et al. (2017) versus proteins with decreased phosphopeptide abundance in the three other datasets. **(E)** Converse comparison of proteins with decreased abundance phosphopeptides in Bhaskara et al. (2017) versus proteins with increased phosphopeptide abundance in the three other datasets. **(F)** Transient expression of candidate proteins identified by Bhaskara et al. (2017) as having putative stress-induced phosphorylation. Each protein was expressed as a fusion with EYFP. All three proteins showed subcellular localization consistent with their predicted localization: nucleus for HMGB1; cytoplasmic for AT5G05600 and endomembrane for Annexin1. All panels show the merged image of EYFP (indicated by green color), chlorophyll fluorescence (indicated by red color) and bright field. Scale bars indicate 10 μm. **(G)** Phos-tag Gel and SDS-PAGE analysis of transiently expressed proteins followed by western blotting with anti-GFP. For each protein, Phos-tag gel separation followed by immunoblotting (upper images) detected increased abundance of slowly migrating bands in samples from stress-treated plants. This was consistent with low water potential-induced phosphorylation. SDS-PAGE followed by immunoblotting (lower images) confirmed the expression of a single form of each protein having the expected molecular weight of the native protein plus the 27 kd of EYFP (total molecular weight of 69 kD for AT5G05600, 63 kD for Annexin1 and 48 kD for HMGB1) and the equal loading of protein for control and stress treated samples. C, unstressed control; S, low water potential stress (–1.2 MPa for 96 h).

There is an increasing realization in drought research that the factors involved in short term acute responses to severe dehydration or osmotic stress differ dramatically from the factors involved in longer term acclimation phenotypes such as growth and metabolic regulation (Skirycz and Inze, 2010; Skirycz et al., 2011; Baerenfaller et al., 2012; Verslues, 2017). Arabidopsis seedlings exposed to -1.2 MPa low water potential stress (essentially a simulated drought treatment using PEG-infused agar plates) for 96 h had up regulation of more than 60 and down regulation of nearly 90 genes encoding kinases and phosphatases (Bhaskara et al., 2012). Baerenfaller et al. (2012) also found that leaf gene expression and proteome composition of plants held at a constant reduced water potential differed dramatically from responses to severe short term stress and recent transcriptome studies have reached a similar conclusion (Des Marais et al., 2012). Results such as these indicate that examining more moderate (or at least non-lethal) low water potential stress over longer time frames (days rather than a few hours) can bring out new aspects of drought response including changes in protein phosphorylation. Several recent studies have examined phosphoproteome remodeling in response to short term (less than 90 min) ABA or dehydration treatment (Kline et al., 2010; Umezawa et al., 2013; Wang et al., 2013; Xue et al., 2013; Stecker et al., 2014; Minkoff et al., 2015). The goal of these studies was to examine the effects of rapid activation or repression of kinase and phosphatase activity before changes in gene expression and protein levels occur (**Figure 1A**). There has been little or no complementary data of phosphoproteome remodeling that occurs over longer term drought acclimation.

We recently reported quantitative phosphoproteome analysis of Arabidopsis plants subjected to longer term (96 h) low water potential stress (Bhaskara et al., 2017). In this case there is sufficient time for stress-induced changes in gene expression and protein levels to occur (**Figure 1A**). Thus, in these longer time frames it is possible to examine the function of the drought-induced kinome (and phosphatome). Here, we discuss comparison of this data set to other published data sets examining short term ABA, dehydration or osmotic stress treatments (**Figure 1A**). These comparisons found little or no overlap between the datasets. There are several reasons for this limited degree of overlap and it shows how we are just beginning to scratch the surface of protein phosphorylation changes and post-transcriptional regulation of protein abundance during drought stress. Such comparison also brought to the fore the continuing challenge of validating the results of large scale phosphoproteomics. We used several phosphoproteins identified in Bhaskara et al. (2017) as test cases to evaluate the use of transient expression and phostag gel analysis for further exploration of putative drought-regulated phosphoproteins.

## Limited Overlap Between Phosphoproteome Changes During Low Water Potential Acclimation Versus Short Term Osmotic Stress or ABA Treatment

The lists of proteins having a significant (*p* ≥ 0.05 and fold change ≥ 1.5) increase or decrease in phosphopeptide abundance in Bhaskara et al. (2017) were compared to three data sets which examined short term responses to dehydration, osmotic stress or ABA (Umezawa et al., 2013; Wang et al., 2013; Xue et al., 2013). The total number of phosphoproteins identified (1500–2500) as well as the number of phosphopeptide abundance changes which could be quantified was roughly similar among all these studies thus facilitating comparison of these data sets. There was no protein with increased phosphopeptide abundance in common between all four datasets, only six (red numbers in **Figure 1B**) in common between three of the four datasets and only ten percent or less overlap between any two of the four studies (**Figure 1B**, lists of phosphoproteins used for comparison are given in Supplementary Table S1). Of the six phosphoproteins increased in three of the four datasets, two were annotated as translation initiation factor-related proteins (**Table 1**). Other than this there was no apparent functional connection between these proteins.

**Table 1:** Phosphorylation sites and functional annotation for selected phosphoproteins found in common between two or more of the four phosphoproteomic data sets compared in **Figures 1B,C**.

Phosphorylation sites^A^					
**Protein**	**Bhaskara**	**Umezawa**	**Xue**	**Wang**	**Description**
**Increased phosphopeptide abundance under stress or ABA treatment**					
AT1G76810	S537 (2.5)^B^	S696	S696	T219	Translation initiation factor 2 (eIF-2) family
			S604 (DOWN)		
AT5G38640	S69 (2.7)^B^	S69	N.R.^C^	S69	NagB/RpiA/CoA transferase-like, Translation initiation factor elF-2B related
	S108 (2.5)	S70			
	S127 (16.5)^B^				
AT4G39680	S292 (1.5)	S415	N.R.	S144	SAP-domain nucleic acid binding protein
		T417	S319
		T421	S324
		S436
		S439
AT5G42950^D^	S1541 (8.6)	S1285	S1520	S1472 (Down)	GYF-domain, Essential for Potexvirus accumulation 1, EXA1
AT1G18210	S88 (1.8)	S54	S54	N.R.	Calcium-binding EF-hand family
		S88
AT2G21230^E^	S300 (1.7)	S174	S174	N.R.	bZIP30
		S176	S176
**Decreased phosphopeptide abundance under stress or ABA treatment**					
AT3G05900	S426 (-2.2) ^B^	S500	S373	S482	Neurofilament protein-related
	S456 (-1.6)	S455
		S456
		S500
AT2G37340	S211 (-1.8)	S202	N.R.	N.R.	Serine/Arginine-rich protein splicing factor RSZ33
	S219 (-1.8)	S204
	S239 (-2.4)	S211
	S245 (-3.5)	S219
AT5G40450	S679 (-3.3)^B^	N.R.	S2802	N.R.	Regulator of vacuole bulb biogenesis1, RBB1
	S2732 (-1.8)
	S2831 (-2.3)^B^
	S2834 (-2.5)
AT4G25880	S30 (-2.1)	N.R.	S154	N.R.	Pumilio (APUM) regulator of mRNA stability and translation

*Phosphoproteins listed here are indicated by red numbers in **Figures 1B,C**. ^A^Phosphorylation site data were obtained from Supplementary Dataset S4 of Bhaskara et al., (2017), Supplementary Table S3 of Umezawa et al. (2013), Supplementary Tables S6, S7 of Xue et al. (2013), and Supplementary Tables S4, S5 of Wang et al. (2013). For several of the proteins listed here, additional phosphorylation sites were identified in one or more of these studies (and can be found in additional supplemental material from those studies) but either did meet their criteria for altered phosphopeptide abundance or were detected but not quantified and are thus not listed here. For Bhaskara et al. (2017) phosphorylation sites having either fold change greater than 1.5 and *P* ≥ 0.05 or fold change greater than 2 regardless of *P*-value are listed. For some of the proteins with up-regulated phosphopeptides in Bhaskara et al. (2017), phosphopeptides having the opposite regulation were also found in other studies and this is indicated by notation of “DOWN” in parentheses. ^B^This difference in phosphopeptide abundance was not significant at *P* ≥ 0.05. ^C^N.R. = Not Reported. No phosphopeptide from this protein was reported to be altered in abundance by stress or ABA treatment. ^D^Phosphopeptide abundance of AT5G42950 with phosphorylation at S39 (Kline et al., 2010) or S749 (Stecker et al., 2014) was also found to be increased by 5 min treatment with 50 μM ABA. ^E^Phosphopeptide abundance of bZIP30 (AT2G21230) with phosphorylation at S176 was also found to be increased by 5 min treatment with 50 μM ABA in Kline et al. (2010) and Minkoff et al. (2015) and found to be increased by NaCl and mannitol (confirmed by SRM assay) in Stecker et al. (2014) Note that the supplemental Data of Minkoff et al. (2015) also includes comparison of their ABA affected phosphorylation sites to those identified in Umezawa et al. (2013) and Wang et al. (2013).*

Particularly surprising was the limited overlap between Xue et al. (2013) and Bhaskara et al. (2017) as both used low water potential or osmotic stress treatments that may at first seem to be similar except for the different time scales. Such statement can also be made about the data of Umezawa et al. (2013), although their up regulated phosphoproteins come from combined data of dehydration and ABA treatments and thus may be expected to be between the stress data of Bhaskara et al. (2017) and that of Wang et al. (2013) which is based solely on ABA treatment. We further examined the phosphorylation sites detected in the six proteins in common between three of the four studies. Of these, only one had the same phosphorylation site detected across three studies while for others different phosphorylation sites were detected (**Table 1**). Thus, there was virtually no overlap in actual up- or down-regulated phosphorylation sites detected in three out of four datasets. That said, two of the proteins in **Table 1** (EXA1 and bZIP30) were also found in studies from the Sussman laboratory (Kline et al., 2010; Stecker et al., 2014; Minkoff et al., 2015) and are of interest for further study as their ABA or stress induced phosphorylation has been repeatedly observed (albeit at different phosphorylation sites).

A similar story emerged when looking at proteins with decreased phosphopeptide abundance. There was only one phosphoprotein in common between all four datasets and only three others in common between Bhaskara et al. (2017) and any of the other three data sets (noted by the red numbers in **Figure 1C**). Indeed, as with the increased phosphoproteins, several of these commonly detected proteins had multiple phosphorylation sites thus increasing their chance to be detected in multiple studies (**Table 1**).

Much of the reason for the limited overlap between the four data sets is technical as each study detected only a small fraction of the thousands of observed or predicted protein phosphorylation sites in Arabidopsis (more than 9000 phosphoproteins and 19,000 unique phosphopeptides are cataloged in PhosPhAt for example^2^). However, this was perhaps not to be the only explanation. To further explore this issue, we did a converse comparison where the proteins with increased phosphopeptide abundance in Bhaskara et al. (2017) were compared to proteins with decreased phosphopeptide abundances in the other three datasets (**Figure 1D**). Interestingly, this converse comparison found greater overlap between up phosphoproteins from Bhaskara et al. (2017) and down phosphoproteins from Xue et al. (2013) than between the up proteins of both studies (13 overlapping proteins in **Figure 1D** compared to one overlapping protein in **Figure 1B**). Such increase in overlapping proteins in the up-versus down comparison was not seen for the phosphoproteins identified by Umezawa et al. (2013) or Wang et al. (2013). This indicated that the lack of congruence between Xue et al. (2013) and Bhaskara et al. (2017) was not just because of technical limitation as a substantial number of the same phosphoproteins were detected but were affected in opposite directions. Part of this may be due to the fact that the data of Bhaskara et al. (2017) can incorporate both changes in protein abundance as well as changes in phosphorylation stoichiometry given the relatively long stress treatment used. However, it should be noted that the 800 mM mannitol treatment used by Xue et al. (2013) is a lethal stress. Possibly this is part of the reason why Xue et al. (2013) found a greater portion of decreased phosphoproteins (181 decreased versus 80 increased) than any of the other three studies which all found more increased phosphoproteins than decreased. The lethality of 800 mM mannitol precludes its use for longer term studies directly comparable to Bhaskara et al. (2017). It has also been noted that mannitol treatment fails to reproduce many drought phenotypes and can instead produce mannitol-specific effects (Verslues et al., 2006; Trontin et al., 2014). Thus Bhaskara et al. (2017) may have confounding effects of changes in protein abundance versus changes in phosphorylation stoichiometry (both of which are drought responses of interest but mechanistically different); however, the data of Xue et al. (2013) also needs to be interpreted cautiously because the up- and down-regulated phosphoproteins they found may reflect cell death responses and the specific effects of mannitol in addition to osmotic stress. It was also interesting to note that there was no overlap between the decreased phosphopeptides of Bhaskara et al. (2017) and the increased phosphopeptides of Xue et al. (2013) (**Figure 1E**).

Another difference between the four phosphoproteomics studies was the criteria used to define increased or decreased phosphopeptides. Both Xue et al. (2013) and Bhaskara et al. (2017) used statistical tests as well as fold-change criterial. Umezawa et al. (2013) and Wang et al. (2013) on the other hand used only a fold-change cutoff with no statistical test. In our experience, many of the largest fold changes in phosphopeptide abundance were variable and did not pass the statistical cutoff (Bhaskara et al., 2017). To see how the different cutoffs affected the overlap between the four phosphoproteomic studies, we compared all phosphoproteins having a 2-fold or greater change in phosphopeptide abundance (no statistical cutoff) in Bhaskara et al. (2017) to the same lists of phosphoproteins from the other three studies (Supplementary Figure S1). Although this included more and different proteins from Bhaskara et al (291 increased and 71 decreased), the results were similar. Particularly there were only a handful of phosphoproteins identified in three of four data sets and less than ten percent overlap between any two data sets (Supplementary Figures S1A,B). Again there was a greater overlap between the increased phosphoproteins of Bhaskara et al. (2017) and decreased phosphoproteins of Xue et al. (2013) (Supplementary Figure S1C). These comparisons in Supplementary Figure S1 did find additional overlapping proteins between the studies that may be of interest for further study as they were identified in more than one study; however, because of the less rigid criteria it becomes even more important to verify the stress-induced phosphorylation of a candidate protein before proceeding with further analysis.

## Seedling Transient Expression Coupled with Phos-Tag Gel Analysis as Strategy to Verify Stress Effects on Phosphorylation

The above discussion illustrates how methods to validate protein phosphorylation and distinguish between changes in phosphorylation stoichiometry versus changes in protein abundance are important complements to large scale phosphoproteome profiling. Such analysis of specific phosphoproteins and phosphorylation sites can be done using either phosphospecific antisera or by selected reaction monitoring (SRM) assays in which a labeled peptide standard is used to develop targeted mass spectrometry detection of a specific phosphopeptide (Maiolica et al., 2012; Stecker et al., 2014). Both of these approaches require the development of specific reagents, the expense of which can be an impediment to exploratory analysis of proteins of unknown or poorly characterized function which predominate in all four datasets discussed here.

As an alternative method to validate and explore the data generated by large scale phosphoproteomics, we tested a combined strategy of transient expression (Tsuda et al., 2012) of YFP-tagged protein combined with Phos-tag gel separation (Kinoshita and Kinoshita-Kikuta, 2011; Kinoshita et al., 2012) and immuno-blotting. By this method we could analyze new phosphoproteins without development of specific reagents for each protein and each phosphorylation site. A key advantage of this system for us was that it used intact Arabidopsis seedlings and allowed us to perform the same 96 h low water potential treatment as used for our original phosphoproteome and transcriptome analysis (Bhaskara et al., 2012, 2017). Three proteins were used as test cases: AT5G05600 (2-oxoglutarate and Fe-dependent oxygenase superfamily protein), Annexin1 (AT1G35720) and HMGB1 (AT3G51880, High Mobility Group B1/Nucleosome and Chromatin Assembly Factor D1). In our phosphoproteomic analysis, two phosphopeptides were found from AT5G05600: one was 35.5-fold more abundant at low water potential while the other was 3.9-fold more abundant. *AT5G05600* gene expression was induced 2.7-fold in the same treatment (Bhaskara et al., 2012, 2017). In this case, the first site may represent a low water potential-induced phosphorylation while the second site could reflect change in protein abundance. Similarly, one phosphopeptide of Annexin1 was 9.1-fold more abundant under stress while another Annexin1 phosphopeptide was 2.4-fold more abundant. *Annexin1* gene expression, however, was increased 4.7-fold by low water potential thus raising some uncertainty as to whether the increased phosphopeptides from Annexin1 were due to stress-induced phosphorylation or change in protein abundance. For HMGB1, two phosphopeptides of 11- and 7-fold increase in abundance at low water potential were identified while *HMGB1* gene expression was not affected by low water potential (Bhaskara et al., 2017). These three proteins also exemplified the limited overlap between phosphoproteomic studies discussed here. Phosphopeptides from AT5G05600 and Annexin1 were decreased by ABA treatment in Wang et al., (2013) and not detected in the other two studies. Similarly, a phosphopeptide from HMGB1 was reported to be decreased by mannitol treatment in Xue et al. (2013) while no HMGB1 phosphopeptides were found to be affected by dehydration or ABA treatment in Umezawa et al. (2013) or Wang et al. (2013).

In transient expression assays, each of these proteins exhibited their expected subcellular localization (**Figure 1F**) and ran as a single band of similar intensity in control and stress treatments on standard SDS-PAGE (**Figure 1G**, bottom panel), consistent with the 35S-driven expression vector used. For each of these three proteins, an upshifted band (or perhaps multiple bands in the case of AT5G05600) consistent with phosphorylated protein, was seen on Phos-Tag gels (**Figure 1G**). For AT5G05600 and Annexin1, the upshifted band was dramatically increased by stress treatment, consistent with the phosphoproteomics data. For HMGB1, the upshifted band was also increased in abundance after stress treatment, although the difference was not as dramatic. As all of these proteins have multiple phosphorylation sites, either from our analysis or from literature (Stemmer et al., 2003), an advantage of the transient expression/Phos-tag analysis is that phosphonull versions of the protein can be expressed and the stress-affected phosphorylation site verified without the commitment of developing phosphospecific antisera or SRM assays for each site. Also, the effects of low water potential stress versus treatment with exogenous ABA could be compared. A disadvantage of this system is that the Phos-Tag gel conditions often need to be optimized for each protein. In particular, highly basic proteins, such as many of the DNA binding and RNA-splicing associated proteins found in our data (Bhaskara et al., 2017) require different Phos-tag conditions. High molecular weight proteins (greater than ∼85 kD) may also be problematic for Phos-tag gels and in these cases the YFP tag may be unsuitable and a smaller epitope tag used instead. It may also be possible in some cases that the fusion to a relatively large tag like YFP could mask some phosphorylation sites. This could also possibly be alleviated by use of a smaller epitope tag. Use of the Avr-Pto system for transient expression may not be appropriate for studies of pathogen response. Despite these cautions, our initial experiments found that combined transient expression and Phos-tag gel analysis is a viable alternative for analysis of new phosphoproteins and phosphorylation sites. This approach is particularly applicable to our analysis of phosphorylation changes associated with longer term drought stress where conducting the analysis in intact plants subjected to reproducible low water potential over the course of several days is critical.

## Conclusion

There is a compelling logic to short term phosphoproteomic analyses which aim to find the initial phosphorylation events that respond most rapidly to altered water status or ABA. There is an equally compelling logic to analyzing longer term treatments where a different set of phosphatases and kinases are expressed. Comparison of four ABA and abiotic stress-related phosphoproteomic studies emphasized how both approaches give complementary, but differing, views of the stress-phosphoproteome. The almost non-existent overlap of stress-regulated phosphorylation sites discovered by each of these studies shows how we have only begun to scratch the surface of the stress phosphoproteome. We also found that a combined transient expression and Phos-tag gel approach is a viable approach to validate and investigate protein phosphorylation. This method can be used in the first steps of investigating unknown or poorly annotated proteins which often come up in phosphoproteomic (and other ‘omic) data. Whether or not one chooses to use this approach or other approaches such as SRM assays, which are becoming more accessible, ultimately depends on the goals and expertise of a given lab and other factors such as accessibility of instrument time. In either case, such protein-level studies are a much needed complement to transcriptome based analyses and promise to reveal new and critical aspects of signaling and protein function required for drought resistance.

## Materials and Methods

*AT3G51880* and *AT5G05600* were cloned into pGWB441 (C-terminal YFP) and *Annexin1* cloned into pGWB442 (N-terminal YFP) (Nakagawa et al., 2007). Transient expression used Avr-Pto seedlings (Tsuda et al., 2012) and modifications to their method to enable seedling expression as previously described (Kumar et al., 2015; Bhaskara et al., 2017). At 96 h after transfer to control or stress plates (5 days after infiltration), seedlings were ground in liquid nitrogen and extracted using lysis buffer consisting of 1% SDS, 10% glycerol, 120 mM Tris-HCl, pH 8.8, Complete protease inhibitor cocktail EDTA-free (Roche), and PhosSTOP Phosphatase inhibitor cocktail (Roche). Protein concentration was quantified by BCA protein assay. Equal amounts of total protein were denatured in 10X loading buffer, separated on 10% SDS-PAGE gels and transferred to PVDF membrane. Immunobloting was performed using anti-GFP primary antibody (Abcam) and anti-rabbit IgG-HRP secondary antibody and signal was developed using Supersignal West Femto Maximum Sensitivity Substrate (Thermo). Phos-tag gel analysis was performed using Zn^2+^-Phos-tag PAGE with neutral-pH gel system (Kinoshita and Kinoshita-Kikuta, 2011). The separating gel consisted of 6% acrylamide, 50 μM Phos-tag acrylamide (Wako), 357 mM bis-tris buffer pH 6.8, 100 μM Zn(NO_3_)_2_. After electrophoresis, the gel was washed using 25 mM Tris, 192 mM glycine, 10% methanol, 1 mM EDTA followed by a second wash with the same buffer lacking EDTA. Immunoblotting was then performed as described above.

## Author Contributions

GB and TN collected and analyzed phosphoproteomics data. T-HY performed transient expression and Phos-tag gel experiments. PV prepared the manuscript and figures with assistance from GB and TN.

## Conflict of Interest Statement

The authors declare that the research was conducted in the absence of any commercial or financial relationships that could be construed as a potential conflict of interest.

## Footnotes

1 http://phosphat.uni-hohenheim.de/statistics.html

2 http://phosphat.uni-hohenheim.de/statistics.html
