# Supplementary material for: Comparative Analysis of Phosphoproteome Remodeling After Short Term Water Stress and ABA Treatments versus Longer Term Water Stress Acclimation
Source: Front Plant Sci. 2017 Apr 11;8:523. doi: 10.3389/fpls.2017.00523 (PMC5386979; doi:10.3389/fpls.2017.00523)
Supplement: Supplementary file 2 [file Image_1.PDF]

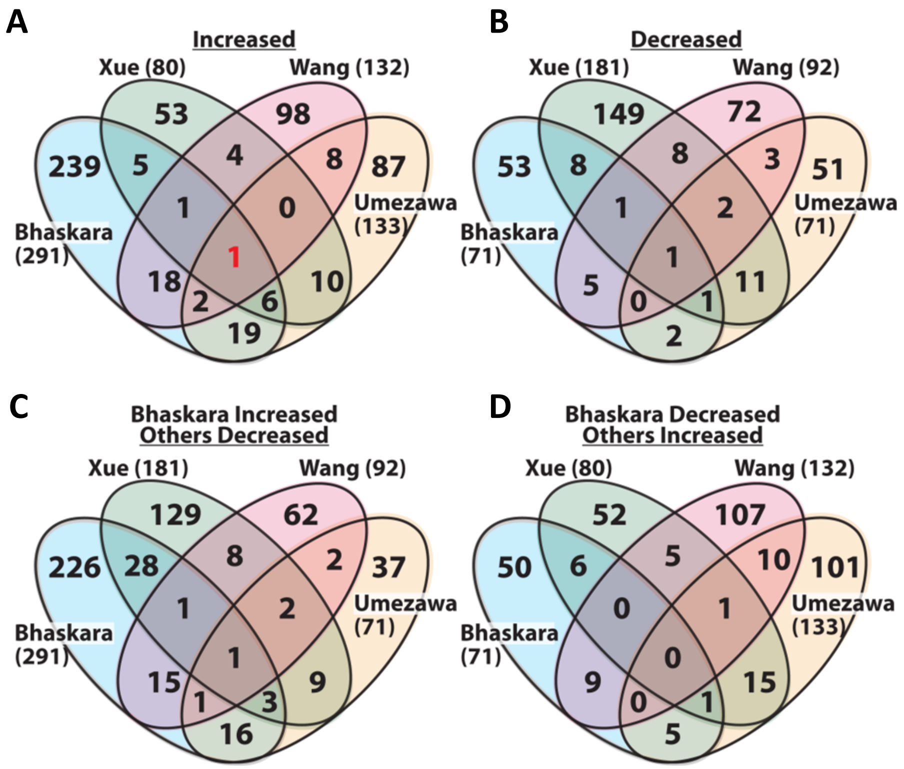

Supplemental Figure S1: Comparative analysis of four abiotic stress and ABA-related phosphoproteomic datasets using all phosphopeptides with two-fold or greater abundance change (no statistical cutoff) in Bhaskara et al. (2017).

A. Comparison of proteins with one or more phosphopeptides of increased abundance in stress or ABA treatment for all four datasets. Each data set is identified by the name of the first author and numbers in parentheses are the total number of proteins with increased phosphopeptide abundance in that dataset. Red number in A indicates that this phosphoprotein is included in Table 1.
B. Comparison of proteins with one or more phosphopeptides of decreased abundance in stress or ABA treatment for all four datasets.
C. Converse comparison of proteins with increased abundance phosphopeptides in Bhaskara et al. (2017) versus proteins with decreased abundance phosphopeptides in the three other datasets.
D. Comparison of proteins with decreased abundance phosphopeptides in Bhaskara et al. (2017) versus proteins with increased phosphopeptide abundance in the three other datasets.
